# Pathologic tearfulness after limbic encephalitis: A novel disorder and its neural basis

**DOI:** 10.1212/WNL.0000000000008934

**Published:** 2020-03-24

**Authors:** Georgios P.D. Argyropoulos, Lauren Moore, Clare Loane, Adriana Roca-Fernandez, Carmen Lage-Martinez, Oana Gurau, Sarosh R. Irani, Adam Zeman, Christopher R. Butler

**Affiliations:** From the Memory Research Group (G.P.D.A., L.M., C.L., A.R.-F., C.L.-M., O.G., C.R.B.) and Autoimmune Neurology Group (S.R.I.), Nuffield Department of Clinical Neurosciences, University of Oxford; Department of Psychology (L.M.), University of Bath; Maurice Wohl Clinical Neuroscience Institute, Basic and Clinical Neuroscience Department (C.L.), King's College London, UK; Valdecilla Biomedical Research Institute (C.L.-M.), University Hospital Marqués de Valdecilla, Santander, Spain; Medical School (A.Z.), University of Exeter, UK; Department of Brain Sciences (C.R.B.) Imperial College London, UK; and Departamento de Neurología (C.R.B.), Pontificia Universidad Católica de Chile, Santiago.

## Abstract

**Objective:**

We investigated the nature and neural foundations of pathologic tearfulness in a uniquely large cohort of patients who had presented with autoimmune limbic encephalitis (aLE).

**Methods:**

We recruited 38 patients (26 men, 12 women; median age 63.06 years; interquartile range [IQR] 16.06 years) in the postacute phase of aLE who completed questionnaires probing emotion regulation. All patients underwent structural/functional MRI postacutely, along with 67 age- and sex-matched healthy controls (40 men, 27 women; median age 64.70 years; IQR 19.87 years). We investigated correlations of questionnaire scores with demographic, clinical, neuropsychological, and brain imaging data across patients. We also compared patients diagnosed with pathologic tearfulness and those without, along with healthy controls, on gray matter volume, resting-state functional connectivity, and activity.

**Results:**

Pathologic tearfulness was reported by 50% of the patients, while no patient reported pathologic laughing. It was not associated with depression, impulsiveness, memory impairment, executive dysfunction in the postacute phase, or amygdalar abnormalities in the acute phase. It correlated with changes in specific emotional brain networks: volume reduction in the right anterior hippocampus, left fusiform gyrus, and cerebellum, abnormal hippocampal resting-state functional connectivity with the posteromedial cortex and right middle frontal gyrus, and abnormal hemodynamic activity in the left fusiform gyrus, right inferior parietal lobule, and ventral pons.

**Conclusions:**

Pathologic tearfulness is common following aLE, is not a manifestation of other neuropsychiatric features, and reflects abnormalities in networks of emotion regulation beyond the acute hippocampal focus. The condition, which may also be present in other neurologic disorders, provides novel insights into the neural basis of affective control and its dysfunction in disease.

Most neurologic research on emotion dysregulation focuses on pseudobulbar affect, which occurs in a broad range of disorders with diffuse or poorly characterized pathology^1–5^ often implicating the brainstem and cerebellum.^1,6–8^ The fact that pseudobulbar affect has not been associated with focal limbic damage is consistent with its being understood as “a disorder of emotional expression rather than a primary disturbance of feelings.”^7^

Autoimmune limbic encephalitis (aLE) is associated with the subacute onset of amnesia and seizures and high T2-signal (acute MRI) in the limbic system, especially the hippocampus. Patients often respond satisfactorily to immunosuppressive therapy,^9^ although many develop hippocampal atrophy and residual cognitive impairment.^10,11^ While behavioral/psychiatric symptoms may occur acutely,^12^ persisting problems with readily provoked tearfulness are only mentioned in passing,^13–15^ and we have encountered complaints of such symptoms among many of our patients.

We aimed to determine the so far unexplored nature and neural correlates of pathologic tearfulness following aLE in a uniquely large cohort of patients (n = 38). We investigated its relationships with demographic and clinical data, self-reported measures of emotion regulation, and performance on neuropsychological tests. We hypothesized that it is associated with abnormalities in the hippocampus, the amygdala, hippocampal-diencephalic-cingulate networks, and cerebro-ponto-cerebellar loops: aLE results in relatively focal hippocampal atrophy,^11,16^ and the limbic system is involved in emotion processing.^17–19^ Amygdala abnormalities are sometimes observed^20^ and have been associated with abnormal autonomic arousal.^21^ Furthermore, the hippocampus is embedded within broader hippocampal-diencephalic-cingulate networks supporting emotion regulation.^22^ We have recently shown abnormalities in this extended circuitry in aLE.^23,24^ Finally, in a prominent pathophysiologic account, emotion dysregulation in pseudobulbar affect was caused by disruption to cerebro-ponto-cerebellar pathways,^7^ with which the hippocampus communicates.^25^

## Methods

### Standard protocol approvals, registrations, and patient consents

Ethical approval was received from the South Central Oxford Research Ethics Committee (REC no. 08/H0606/133). All participants provided written informed consent according to the Declaration of Helsinki.

### Participants

We report data relating to pathologic tearfulness in 38 patients with aLE (26 male, 12 female; median age at research MRI 63.06 years; interquartile range [IQR] 16.16 years)^24^ after the acute stage of the disease (median 5.41; IQR 5.36 years since symptom onset). All patients were fluent in English (37 native speakers; 1 non-native speaker) and had undergone MRI at the time of initial clinical presentation as well as neuropsychological assessment at the Russell Cairns Unit, Oxford, UK (2013–2018).

All patients had been diagnosed with aLE according to established diagnostic criteria^26^: (a) subacute symptom onset suggesting involvement of the limbic system; (b) bilateral abnormalities restricted within the medial temporal lobes (MTLs) on T2-weighted MRI; (c) CSF pleocytosis (white blood cells >5/mm^3^) or slow-wave/epileptic activity involving the temporal cortex (EEG); (d) exclusion of alternative causes (e.g., CNS infections/drug toxicity/stroke/Creutzfeldt-Jakob disease/Kleine-Levin syndrome, mitochondrial/neoplastic/epileptic/rheumatologic disorders, septic/metabolic encephalopathy); (e) antibodies against cell-surface/synaptic/onconeural proteins. Criteria (a–d) are required for a diagnosis of definite limbic encephalitis, unless, in the absence of one of (a–c), criterion (e) is satisfied.^26^

A total of 34 of 38 patients satisfied the criteria for a diagnosis of definite aLE; the remaining 4/38 had been diagnosed with aLE, meeting criteria (a, b, d), but not (e). No data could be recovered regarding (c). In 28/38 patients, an aLE-associated autoantibody was identified. A total of 10/38 patients demonstrated the clinical profile of aLE with no identified antibody; such cases are well-recognized^27^ and are generally thought to involve antibodies not detected in clinical practice at the time of screening. No patient presented with positive PCR testing for herpes simplex virus or with anti-NMDAR encephalitis.^28^ Two of 38 patients had neoplastic lesions, thought to be the triggers for their autoimmune disorder, which were treated and were in full remission at the time of study participation. A total of 31/38 patients had been treated acutely with immunotherapy (e.g., plasma exchange, IV or oral prednisolone). A total of 34/38 patients had shown abnormal hippocampal signal, volume, or diffusion on clinical MRI conducted acutely. Six of 38 patients showed amygdala abnormalities, 1 in the parahippocampal cortex, 1 in the entorhinal cortex, 4 patients had mild microangiopathic changes in keeping with their age, and 1 patient showed extra-MTL abnormalities (bright caudate). No acute abnormalities were detected in 4/38 patients, who nonetheless demonstrated clinical features characteristic of aLE; 35/38 patients had presented acutely with seizures.

Moreover, patients had no history of previous neurologic or psychiatric disorder that could have resulted in cognitive impairment. They were assessed by a single neurologist (CRB) prior to study inclusion. Their (acute) clinical and (postacute) neuropsychological details have been presented previously.^24^ Healthy controls (HCs) were recruited through the Oxford Project to Investigate Memory and Ageing (OPTIMA) and through local advertisement.

### Neuropsychological profile

Postacutely, all patients and 57 HCs (38 men, 19 women; age at assessment: median, 61.50; IQR 17.26 years; HCs vs patients: male:female ratio: χ^2^ = 0.032, *p* = 0.858; age at assessment: *U* = 933.50, *p* = 0.258) underwent neuropsychological assessment. Patients showed preserved executive function, above-average premorbid intelligence, and spared motor, executive, and visuospatial function, but impaired episodic memory.^24^

### Review of medical records

Details were extracted from medical records and interviews with the patients and caregivers using a standard proforma regarding clinical history, acute aLE presentation, and subsequent clinical course of each patient (age at symptom onset; presenting symptoms; premorbid and acute phase depression, anxiety, agitation, obsessionality, or hallucinations; seizure occurrence/recency; delay between symptom onset and start of treatment; autoantibody type; past/present immunotherapy, antiepileptics, and antidepressants).

### Emotion regulation assessment

#### Questionnaires

In order to assess patients' pathologic tearfulness, we administered the Center for Neurologic Study–Lability Scale (CNS-LS),^29^ a 7-item questionnaire comprising 2 subscales (“labile crying” and “labile laughter”). A series of additional questionnaires were administered to examine the relationship of patients' pathologic tearfulness with (1) anxiety and depression (Hospital Anxiety and Depression Scale [HADS]),^30^ (2) impulsivity (Barratt Impulsiveness Scale [BIS]),^31^ (3) irritability (Irritability Questionnaire [IRQ]),^32^ and (4) empathy (Cambridge Behaviour Scale [CBS]^33^; docs.autismresearchcentre.com/tests/EQ40_ScoringKey.doc). A total of 25/38 patients and 29/57 HCs completed and returned those self-administered questionnaires by post. Patients filled out the questionnaires together with their next of kin or family members. Patients who completed the emotion regulation questionnaires did not differ from those who did not in the following: (1) neuropsychological tests in which patients showed preserved group-level performance^24^ (all *p*s, *p*_corr_ ≥0.340); (2) tests in which patients showed group-level impairment^24^ (all *p*s, *p*_corr_ ≥0.304); (3) clinical/demographic variables (see previous section; all *p*s, *p*_corr_ ≥0.999); (4) volumes of manually delineated MTL structures and automatically delineated subcortical structures in which there was no group-level atrophy^24^ (all *p*s, *p*_corr_ ≥0.209); and (5) structural/functional brain abnormalities identified at group level^24^ (all *p*s, *p*_corr_ ≥0.260).

We also assessed the relationship of patients' emotion regulation with their memory by conducting bivariate correlation analyses between memory test scores and scores on questionnaires of emotion regulation in which patients showed impairment compared with HCs.

#### Self-report (clinical interview)

In a complementary approach, and since the CNS-LS may not be sensitive to the symptoms described by our patients, we dichotomized the cohort according to clinical complaint at interview. The interviewer was blind to patients' responses in the above questionnaires. Patients and their family members were asked whether there had been instances of “labile laughter” or labile crying, and to provide examples from their daily life.

#### Relationship with demographic, clinical, and neuropsychological profiles

We conducted (1) bivariate correlations of CNS-LS scores with continuous variables and independent-samples comparisons on CNS-LS scores for binary variables across patients; and (2) comparisons among HCs, patients with, and patients without pathologic tearfulness (independent-samples comparisons for continuous variables, χ^2^ tests for binary variables).

### Brain imaging

#### Structural MRI

We acquired 3D T1-weighted images using a magnetization-prepared rapid gradient echo sequence (echo time 4.7 ms, repetition time 2,040 ms, 8° flip angle, field of view 192 mm, voxel size 1 × 1 × 1 mm). All 38 patients (26 male, 12 female; age at imaging: median 63.06; IQR 16.06 years) underwent structural brain imaging, along with 67 HCs (35 recruited by the Memory and Amnesia Project [MAP]; 32 datasets were made available through OPTIMA; 40 male, 27 female; age at imaging: median 64.70; IQR 19.87 years; HCs vs patients: M:F ratio: χ^2^ = 0.79, *p* = 0.374; age at imaging: *U* = 1,239.5; *p* = 0.825) (methods also in reference 24).

#### Volumetry

MTL structures (left/right hippocampus, amygdala, temporopolar, entorhinal, perirhinal, and parahippocampal cortices) were manually delineated in native space (protocol: ndcn.ox.ac.uk/files/research/segmentation_protocol_medial_temporal_lobes.pdf).^23,24^ Subcortical structures (brainstem, left/right thalamus, caudate nucleus, putamen, pallidum, nucleus accumbens) were automatically delineated using FSL-FIRST (v.6.0; https://fsl.fmrib.ox.ac.uk/fsl/fslwiki).^34^

#### Whole-brain voxel-based morphometry (VBM)

In order to identify gray matter (GM) volume reduction in our patient group at a whole-brain level, the T1-weighted MRIs were analyzed with VBM, conducted using Statistical Parametric Mapping software (SPM12; fil.ion.ucl.ac.uk/spm/software/spm12) in MATLAB R2017b. Images were examined for scanner artefacts and reoriented to have the same point of origin (anterior commissure) and spatial orientation. They were then bias-corrected to remove intensity nonuniformities, and segmented into GM, white matter (WM), and CSF with the unified segmentation procedure. The diffeomorphic anatomical registration through the exponentiated lie algebra (DARTEL) toolbox was applied to participants' GM, WM, and CSF to refine intersubject registration, and study-specific GM templates were generated.^35^ After affine registration of the GM DARTEL templates to the tissue probability maps in Montreal Neurologic Institute (MNI) space, nonlinear warping of GM images was performed to this template in MNI space. Voxel values in the tissue maps were modulated by the Jacobian determinant (calculated during spatial normalization), with modulated GM images reflecting tissue volume. These images (voxel size: 1 mm^3^ isotropic) were smoothed using a Gaussian filter of 8 mm full width at half maximum (FWHM). We compared GM volume between groups (HCs > patients; between-subject covariates: age, sex, total intracranial volume [TIV], study [MAP, OPTIMA]). We report clusters surviving family-wise error (FWE) correction (*p* < 0.05) at peak voxel level over *p* < 0.001 (uncorrected), as well as clusters surviving correction for nonstationary smoothness^36^ and FWE correction for cluster size (*p* < 0.05).

Volumes (calculated from manual/automated segmentation, or the volume reflected by each VBM cluster) that showed reduction in patients at whole-group level were residualized against age, sex, TIV, and study and entered in bivariate correlation analyses with scores in questionnaires of emotion regulation. We also contrasted patients with pathologic tearfulness with those without and HCs across all volumes delineated as well as across the whole brain (VBM).

#### Resting-state fMRI (rsfMRI)

Whole-brain blood oxygenation level–dependent (BOLD)–weighted fMRI data were acquired (gradient echo echoplanar imaging (EPI) sequence; 180 volumes; slice thickness 3.5 mm, echo time 28 ms, repetition time 2,410 ms, 89° flip angle, field of view 192 mm, voxel size 3 × 3 × 3.5 mm). Participants were instructed to lie still, not to fall asleep, to keep their eyes open, and to watch a fixation cross presented on the in-scanner projector. A total of 35 of 38 patients (3 datasets discarded due to acquisition errors or movement; 24 men, 11 women; median age at imaging, 61.45; IQR 15.85 years) underwent rsfMRI, along with 32 HCs (3 datasets discarded due to movement or acquisition errors; only structural MRIs were available for the HCs that were made available through OPTIMA; 23 men, 9 women; median age 55.71; IQR 17.18 years; HCs vs patients: male:female ratio: χ^2^ = 0.087; *p* = 0.768; age at imaging: *U* = 425.00; *p* = 0.091).

#### Preprocessing

EPIs were spatially realigned and slice time–corrected. Structural MRIs were coregistered to the EPIs, segmented and normalized along with EPIs in MNI space, followed by motion outlier detection (artifact detection tools–based scrubbing). Denoising, including the anatomical component-based correction method (CompCor), was employed to remove sources of noise in the BOLD time series data, deriving principal components from WM and CSF. WM, CSF, and the 6 movement measures were included as first-level nuisance covariates. A temporal bandpass filter (0.01–0.1 Hz) was applied to this residual BOLD signal, in order to remove motion artefacts and physiologic and other artefactual effects. Images were smoothed using a Gaussian filter (8 mm FWHM).

### Resting-state amplitude of low frequency fluctuations and functional connectivity

We further examined whether resting-state abnormalities in the local amplitude of low-frequency fluctuations (rsALFF) and hippocampal functional connectivity (rsFC) were associated with pathologic tearfulness. Preprocessing, rsALFF, and rsFC analyses were conducted using the CONN toolbox v. 18.a (nitrc.org/projects/conn).^37^

#### rsFC: connectome–multivariate pattern analysis (MVPA)

In order to identify seed regions for post hoc seed-to-voxel connectivity analyses in a data-driven fashion, we used MVPA as implemented in the connectome-MVPA CONN toolbox. MVPA assesses the multivariate pattern of pairwise connections between voxels across the entire brain by means of a principal component analysis (PCA) separately for each voxel that characterizes its rsFC with the rest of the brain. In the first PCA step, separately for each participant, a default number of 64 PCA components were retained while characterizing each participant's voxel-to-voxel correlation structure. The resulting component scores were stored as first-level voxel-to-voxel covariance matrices for each participant. In the second PCA step, separately for each voxel and jointly across participants, the 7 strongest components were retained from a PCA decomposition of the between-subjects variability in seed-to-voxel connectivity maps between this voxel and the rest of the brain, according to a conventionally employed ratio of 1:10 between the number of components extracted and the number of participants (n = 67). Second-level analyses were then conducted in order to test for group differences in whole-brain connectivity (*F* test across all MVPA components), comparing for each voxel the component scores between the 2 groups (HCs < > patients; between-subjects covariates: age, sex). The results for each voxel reflected between-group differences in rsFC between this voxel and the rest of the brain.

#### rsFC: seed-to-voxel connectivity analysis

We followed up the MVPA with post hoc analyses to determine specific connectivity patterns. We thus conducted a whole-brain seed-to-voxel analysis, seeding from the regions identified from the MVPA contrast (HCs < > patients), in order to assess connectivity between those regions and the rest of the brain.

#### Resting-state hemodynamic activity: rsALFF

Along with rsFC, we also examined local abnormalities in the intensity of slow spontaneous fluctuations of hemodynamic activity at rest across the whole brain, using an analysis of rsALFF, that is, the total power within the frequency range between 0.01 and 0.1 Hz, indexing the strength of low-frequency oscillations.

All the rsfMRI analyses involved age and sex as between-subjects covariates. Statistical parametrical connectivity maps were thresholded at a voxel level of *p* < 0.001 and FWE-corrected (*p* < 0.05) at cluster or peak level.

The mean values in clusters of reduced rsALFF or rsFC in patients at whole-group level, as compared with HCs, were residualized against age and sex across participants and then entered in bivariate correlations with scores in questionnaires of emotion regulation. We also contrasted patients with pathologic tearfulness against the rest of the patients and HCs across the whole brain.

### Statistical analysis

Statistical (nonimaging) analyses were conducted using SPSS (v. 25.0, SPSS Inc., Chicago, IL). Significance values were corrected for multiple testing with the Holm-Bonferroni sequential correction method (*p*_corr_). We used the Levene test to assess variance homogeneity and the Shapiro-Wilk test to assess normal distribution. When normal distribution was violated (and log-transformation did not suffice), nonparametric tests were employed. Parametric (Student or Welch *t* tests) and nonparametric tests (Mann-Whitney *U*) were used appropriately for independent-samples comparisons. For comparisons among 3 groups, univariate analyses of variance (ANOVAs) or Kruskal-Wallis *H* tests were used appropriately, and post hoc comparisons between groups were Bonferroni-corrected. Pearson *r* and Spearman ρ were used appropriately to examine correlations between questionnaire scores and other measures of interest. We used multiple stepwise linear regression analysis (default α level of 0.05 for entry to model and 0.1 for removal) to assess the proportion of the variance of patients' scores (questionnaires on emotion regulation) that could be explained by brain abnormalities.

### Data availability

The deidentified data will be available and shared by request for purposes of replicating procedures and results.

## Results

### Emotion regulation assessment

#### Questionnaires: patients vs HCs

Patients scored higher than HCs for labile crying (CNS-LS) (*t* = −2.79, *p*_corr_ = 0.049) but not for laughter (*t* = 0.44, *p*_corr_ >0.999; 2-way mixed-effects ANOVA: group: *F* = 2.49, *p* = 0.12; emotion: *F* = 1.81, *p* = 0.19; group × emotion: *F* = 5.73, *p* = 0.02). They did not differ from HCs in their empathy quotient (CBS) (*t* = 0.79, *p*_corr_ >0.999), in irritability (IRQ) (frequency: *U* = 235, *p*_corr_ = 0.450; intensity: *U* = 240.5, *p*_corr_ = 0.450), or anxiety (HADS: *U* = 517, *p*_corr_ = 0.090). They scored higher in the planning (*t* = −4.97, *p*_corr_ <0.0005) and attention facets (*t* = −3.90, *p*_corr_ = 0.002), but not in the motor facet for impulsiveness (BIS) (*t* = 0.38, *p*_corr_ >0.999). They also scored higher for depression (HADS) (*U* = 357.5, *p*_corr_ < 0.0005), although no patient scored within the severe range (also noted in reference 24).

Scores for labile crying did not correlate across patients with impulsiveness (attention and planning facets: ρ = 0.12, *p* = 0.60), depression (ρ = 0.24, *p* = 0.28), or any memory score in which patients had shown impaired performance as compared with HCs (all *p*s, *p*_corr_ ≥0.240), and were not associated with any demographic or clinical variables examined (all *p*s, *p*_corr_ ≥0.440).

#### Self-report: patients with vs patients without pathologic tearfulness and HCs

In a research-oriented clinical interview, 19 of 38 patients were identified as presenting with pathologic tearfulness. In particular, they reported being moved to tears easily by relatively minor stimuli in a manner at odds with their premorbid state (table 1). The other 19 reported never having experienced such instances. No patient reported experiencing episodes of labile laughter.

**Table 1:**
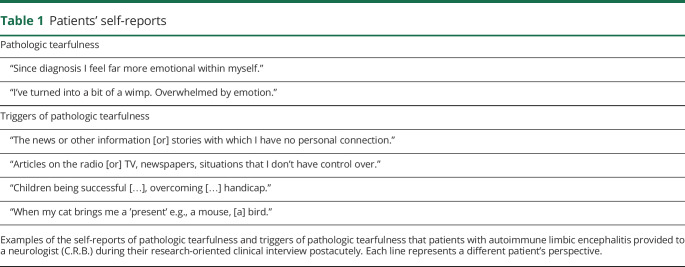
Patients' self-reports

The majority of patients and their family members reported specific triggers of such reactions, including sad stories on the news and witnessing other people crying (table 2).

**Table 2:**
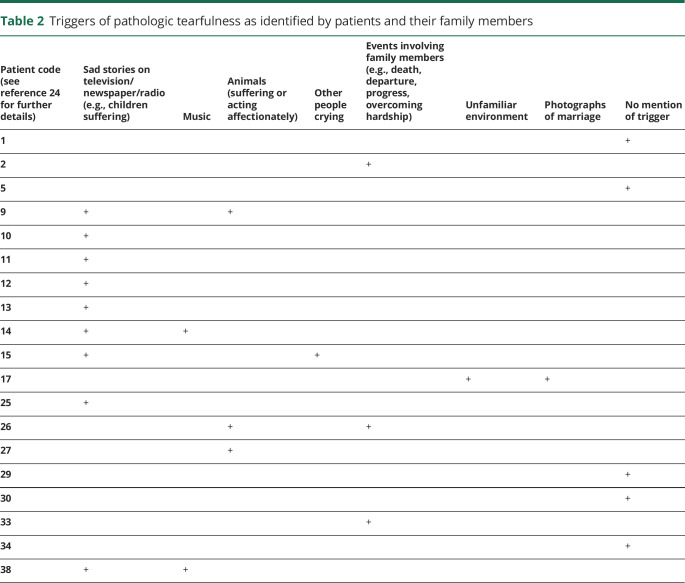
Triggers of pathologic tearfulness as identified by patients and their family members

Patients with pathologic tearfulness did not differ from the rest of the participants in any demographic or clinical details or in episodic memory impairment, depression, or impulsiveness. Moreover, they did not differ from the rest of the patients or HCs in premorbid intelligence, vocabulary, semantic knowledge, visuomotor function or executive function, anxiety, empathy, or irritability. Among all the tests and questionnaires administered, the only one in which they scored differently from both the rest of the patients and HCs was CNS-LS (table 3).

**Table 3:**
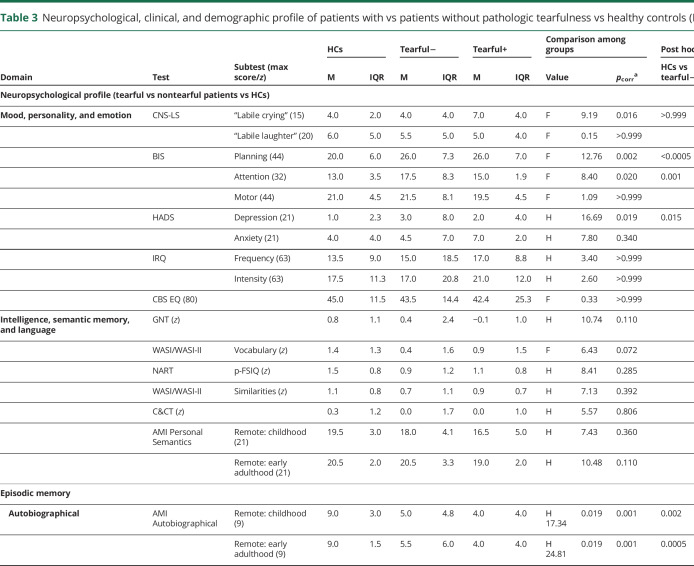

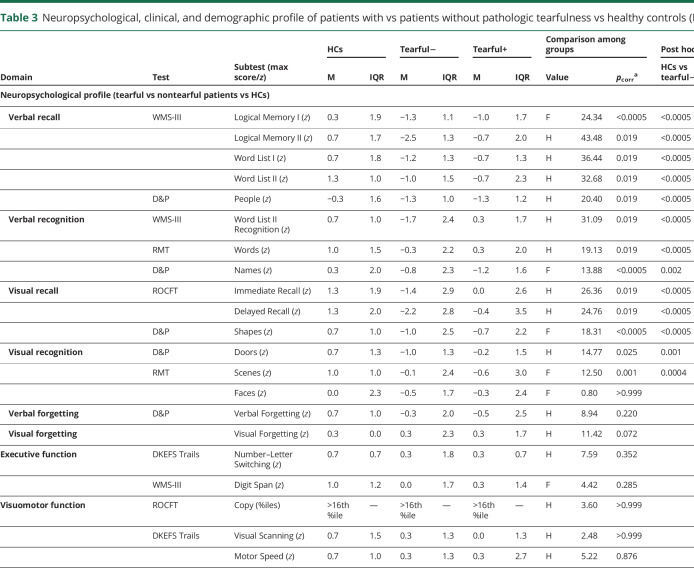

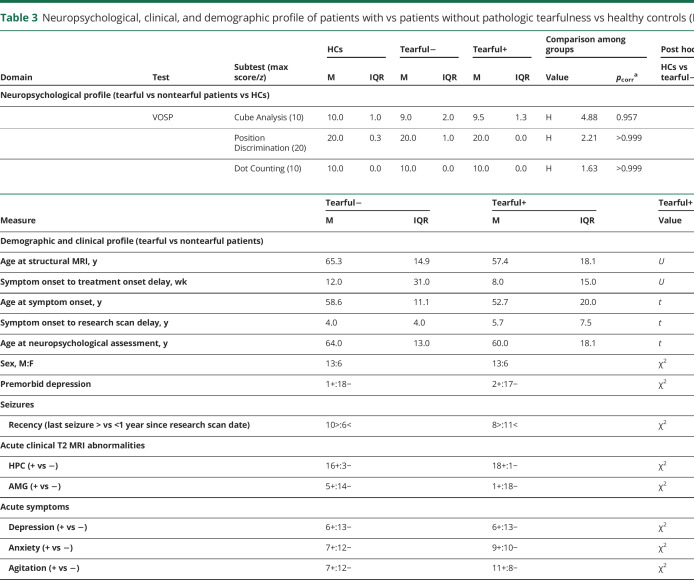

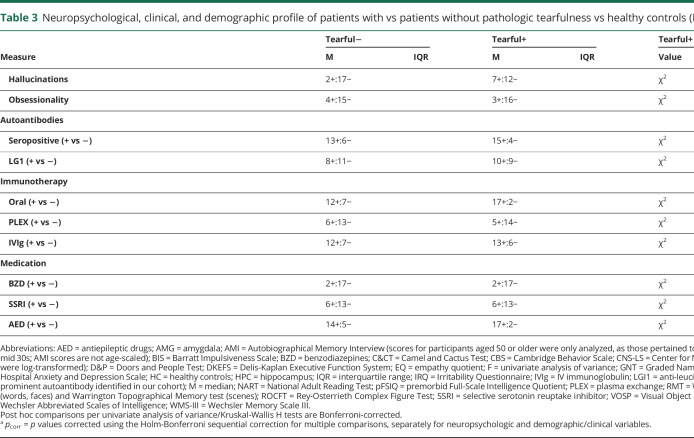
Neuropsychological, clinical, and demographic profile of patients with vs patients without pathologic tearfulness vs healthy controls (HCs)

### Structure/function–Behavior relationships

#### Questionnaires: correlations with CNS-LS and BIS scores

**Figure 1:**
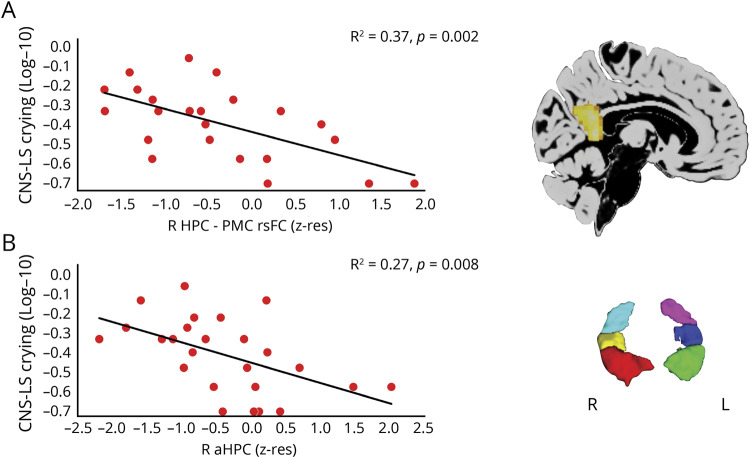
“Labile crying” scores in patients with autoimmune limbic encephalitis: structure/function–behavior relationships Bivariate correlations between Center for Neurologic Study–Lability Scale (CNS-LS) scores for labile crying (log-transformed) and measures of structural and functional abnormalities. (A) Correlation with mean resting-state functional connectivity (rsFC) between the right hippocampus (HPC) and posteromedial cortex (PMC). (B) Correlation with volume of the (manually delineated) right anterior HPC (head). Red = right HPC head; yellow = right HPC body; teal = right HPC tail; green = left HPC head; blue = left HPC body; pink = left HPC tail. R, L = right, left (hemisphere); z-res = volumes are residualized against age, sex, total intracranial volume, and study (Memory and Amnesia Project, Oxford Project to Investigate Memory and Ageing) across participants; mean rsFC values are residualized against age and sex across participants.

In our previous study,^24^ we identified a series of brain abnormalities (n = 13) that patients showed at group level: volume reduction in the left and right hippocampus, captured by both VBM and manual delineation; volume reduction in the anterior-mediodorsal thalamus and right dorsolateral thalamus (VBM) and the left thalamus (automated delineation), as well as the right entorhinal cortex (manual delineation); reduced right hippocampal rsFC with left hippocampus, ventral-posterior posteromedial cortex (posterior cingulate, retrosplenial cortex, and precuneus; Brodmann area [BA] 23, 31), and medial prefrontal cortex (BA 10, 32, 24); and reduced rsALFF in the posterior cingulate and the precuneus (BA 23, 31). We entered the mean values of the clusters that reflected these abnormalities (residualized against age and sex for functional abnormalities, as well as TIV and study [MAP, OPTIMA] for volumes) in bivariate correlations with CNS-LS scores for labile crying. Patients' scores correlated strongly with their reduced right hippocampal rsFC with the ventral-posterior posteromedial cortex (*r* = −0.61, *p*_corr_ = 0.030; rest of *p*s, *p*_corr_ ≥0.190; figure 1A). No such correlations were identified with impulsivity (BIS attention and planning facets) or depression (HADS) scores, even at uncorrected levels (|*r*| <0.29, *p* > 0.18).

Moreover, given our a priori hypotheses on the role of the hippocampus in emotion dysregulation, we examined, at uncorrected levels, correlations with CNS-LS scores. Right anterior hippocampal volume correlated negatively across patients with scores for labile crying (*r* = −0.52, *p* = 0.01; left anterior, right/left posterior hippocampus: *p* > 0.07; figure 1B), but not with right hippocampal rsFC with the posteromedial cortex (*r* = 0.33, *p* = 0.05). When these 2 factors were entered as independent variables in a multiple stepwise linear regression, the analysis was terminated in 2 steps, with the right hippocampal–posteromedial cortical rsFC included in the first model as a predictor of patients' scores of labile crying (*F* = 12.50, *p* = 0.002; *R*^2^ = 0.37), and with the volume of the right anterior hippocampus entered in the model in the second step (*F* = 9.55, *p* = 0.001; *R*^2^ = 0.49). No volumetric correlation of any hippocampal segment was identified with impulsivity or depression scores (|*r*| <0.25, *p* > 0.19).

### Self-report: patients with vs without pathologic tearfulness and HCs

#### Structural abnormalities

**Figure 2:**
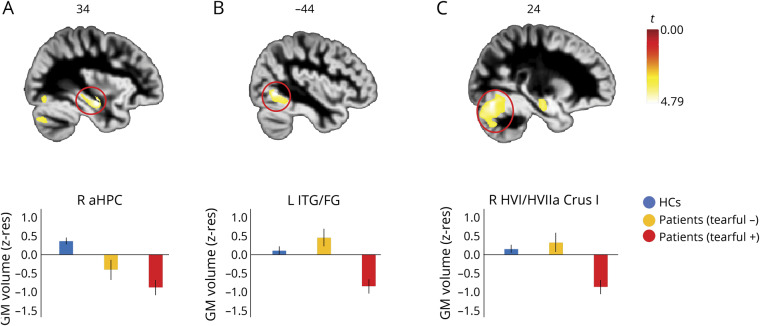
Structural abnormalities in patients with pathologic tearfulness Results of whole-brain voxel-based morphometry (VBM) on modulated gray matter (GM) (reflecting GM volume). Contrast: healthy controls (HCs) and patients without pathologic tearfulness > patients with pathologic tearfulness; between-subjects nuisance regressors: age, sex, total intracranial volume (TIV), and study (Memory and Amnesia Project [MAP], Oxford Project To Investigate Memory and Ageing [OPTIMA]). (A) Right anterior hippocampus: kE = 19, *p* family-wise error-corrected (FWE) = 0.037; peak voxel: *t* = 4.79; x = 34, y = −12, z = −17. (B) Left fusiform gyrus/posterior portion of inferior temporal gyrus: kE = 17; *p* FWE = 0.038; peak voxel: *t* = 4.79; x = −44, y = −62, z = −5. (C) Right cerebellar hemispheric lobules VI/VIIa Crus I: kE = 23; *p* FWE = 0.042; peak voxel: *t* = 4.76; x = 24, y = −75, z = −18; clusters are displayed here at *p* < 0.001 (unc) for display purposes, and survive FWE correction (*p* < 0.05) at peak-voxel level over *p* < 0.001 (unc) (minimum cluster volume: kE > 10). The cerebellar cluster also survived correction for nonstationary smoothness and cluster size (p-FWE < 0.05). Clusters are overlaid here on a diffeomorphic anatomical registration through exponentiated lie algebra GM template in Montreal Neurological Institute space (sagittal sections presented); heat bar represents *t* values; bar graphs display the average GM volume of each of those 3 clusters for the 3 different groups; error bars represent +1/−1 SEM. aHPC = anterior hippocampus; FG = fusiform gyrus; ITG = inferior temporal gyrus; kE = cluster size (number of voxels); R, L = right, left (hemisphere); z-res = mean values residualized against age, sex, study (MAP, OPTIMA), and TIV across participants.

Patients with pathologic tearfulness did not differ from the rest of the patients in any MTL or subcortical volumes (*p*_corr_ ≥ 0.350). Nevertheless, a whole-brain VBM analysis disclosed lower volume for these patients relative to the other 2 groups in the right anterior hippocampus, the right cerebellar hemispheric HVI/HVIIa Crus I, and the left fusiform gyrus (BA 37; figure 2).

#### Functional abnormalities

**Figure 3:**
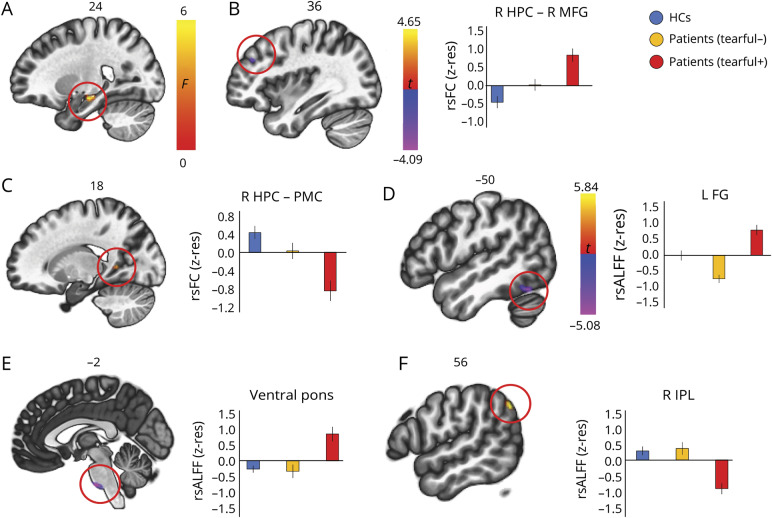
Functional abnormalities in patients with pathologic tearfulness (A) A connectome–multivariate pattern analysis demonstrated that patients with pathologic tearfulness, as compared with the rest of the patients and healthy controls (HCs), showed abnormal resting-state functional connectivity (rsFC) between a region in the right hippocampal (HPC) head and body and the rest of the brain; right hippocampal head and body: 24 −16 −14, kE = 194; *p* family-wise error-corrected (FWE) (cluster-level) = 0.001. (B-C) Patients with pathologic tearfulness showed (B) increased rsFC of the right hippocampus with the right middle frontal gyrus (MFG) (x = 36, y = 40, z = 30; kE = 105, *p* FWE = 0.03, *t* = −4.09), and (C) reduced rsFC of the right hippocampus with the posteromedial cortex (PMC) (peak voxel: *t* = 4.65; x = 18, y = −50, z = 6; kE = 98, *p* FWE = 0.04), extending to the right lingual gyrus. (D–F) Patients with pathologic tearfulness showed aberrantly increased resting-state amplitude of low frequency fluctuations (rsALFF) as compared with both the rest of the patients and healthy controls in (D) the left fusiform gyrus (kE = 69; peak voxel: *t* = −4.72; x = −50, y = −60, z = −20), and (E) the ventral pons (kE = 74; peak voxel: *t* = −5.08; x = −2, y = −20, z = −44; *p* FWE = 0.04), and reduced rsALFF in (F) the right inferior parietal lobule (peak voxel: *t* = 5.84; x = 56, y = −62, z = 40; kE = 112; *p* FWE = 0.004). All clusters survive FWE correction (*p* < 0.05) for cluster size over an uncorrected individual voxel threshold of *p* < 0.001. Error bars represent ± 1 SEM. FG = fusiform gyrus; IPL = inferior parietal lobule; kE = cluster size; R, L = right, left (hemisphere); z-res = mean rsFC and rsALFF values are residualized against age and sex across participants.

A connectome–MVPA analysis on rsFC across the whole brain identified a cluster in the right hippocampus as a region in which patients with pathologic tearfulness differed from the other 2 groups. We thus seeded from the right hippocampus in native space (unsmoothed timeseries), in order to identify regions with which these patients showed abnormal right hippocampal rsFC: they showed aberrantly increased right hippocampal rsFC with the right middle frontal gyrus (BA 9) and reduced rsFC with a region in the right posterior cingulate extending to the precuneus and lingual gyrus (BA 23, 18). Patients with pathologic tearfulness also showed aberrantly increased rsALFF in the left fusiform gyrus (BA 37) and the ventral pons, as well as reduced rsALFF in the right inferior parietal lobule (BA 39; figure 3).

## Discussion

Our study is the first to investigate the nature and neural foundations of emotion dysregulation in a uniquely large, homogeneous cohort of patients after aLE, a nondegenerative neurologic syndrome characterized by primary limbic pathology.

### Clinical features and correlates of emotion dysregulation

In particular, we describe a novel disorder of emotion regulation following aLE that is characterized by residual pathologic tearfulness. In our cohort, this was reported by 50% of patients. This symptom may be misdiagnosed as a manifestation of depression; for example, an indirect consequence of reduced quality of life due to memory impairment. If present alongside disinhibition and impulsiveness, it may otherwise be interpreted as a sign of a broader dysexecutive syndrome, continuous with that sometimes present in the acute stage of aLE.^10^ However, we showed that pathologic tearfulness was not associated with depression or impulsiveness, and occurred in the face of preserved executive function, and at normal levels of anxiety and irritability. Notably, no clinical or behavioral difference was detected between the patients with pathologic tearfulness and the equally sized subset with no such symptoms, apart from their scores on labile crying (CNS-LS).

To our knowledge, this symptom has only been mentioned in passing in case or case series studies of aLE^13–15^ or in studies of larger yet less homogeneous cohorts of autoimmune encephalitis or epilepsy,^38,39^ as “emotional lability,” “mood lability,” or “uncharacteristic tearfulness,” with no further discussion of its clinical features and correlates. Direct comparisons with other patient groups such as temporal lobe epilepsy will be needed in future studies. Moreover, the profile of pathologic tearfulness observed in our patients with aLE is strikingly different from the syndrome of pseudobulbar affect seen in other neurologic conditions (e.g., amyotrophic lateral sclerosis, stroke, multiple sclerosis, Parkinson disease, Alzheimer disease, and traumatic brain injury^1–5^), where dramatic and debilitating bouts of laughing or crying occur often without any appropriately valanced trigger^7^ or congruence between the experience and expression of emotion.^40^ For instance, none of our patients presented with pathologic laughing. Furthermore, most patients who presented with pathologic tearfulness readily identified specific triggers that were congruent with their albeit exaggerated emotional responses. Many of these triggers pertained to situations that evoked empathic concern (e.g., children or animals in distress or acting affectionately). This may suggest that aberrantly increased empathy underlies the patients' symptoms. There are, indeed, strong links between empathy and proneness to crying in the healthy population, which may suggest that increased empathy is associated with increased likelihood to experience distress, resulting in a higher crying proneness (see reference 41). Whereas the CBS did not disclose abnormality in patients with pathologic tearfulness, it may lack sensitivity in capturing increased, rather than decreased, empathy. Likewise, while the CNS-LS represents the most broadly employed self-report measure of affective lability,^29^ more targeted instruments need to be employed, examining autonomic responses within the context of finer-grained behavioral tasks. This might lead to identification of similar symptoms in other neurologic disorders, such as temporal lobe epilepsy, where suggestive evidence has already been presented.^42^

### Structural and functional correlates

**Figure 4:**
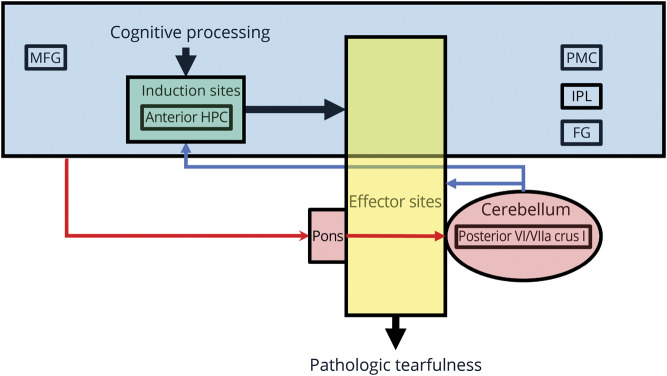
Network abnormalities underlying pathologic tearfulness in autoimmune limbic encephalitis (aLE) Illustration of the network abnormalities that may underlie pathologic tearfulness following aLE, based on our findings and on the model of “pathological laughing and crying” proposed by Parvizi et al. (2001). The locations mentioned in the figure are only those identified as abnormal in this study, and other regions are likely to be involved as well. Blue box = telencephalic sites that are assumed to process “emotionally competent” stimuli along with relevant context information that may include the middle frontal gyrus (MFG), the posterior ventral posteromedial cortex (PMC), the inferior parietal lobule (IPL), and the fusiform gyrus (FG); these act on the induction sites (green box) (e.g., ventromedial prefrontal cortex, anterior cingulate, amygdala, ventral striatum), which may also include the anterior hippocampus (HPC); these sites detect the stimuli and context, and act on the effector sites (yellow box) (e.g., motor cortex, hypothalamus, periaqueductal gray, cranial nerve nuclei), which trigger the emotional response. Red arrows = cerebro-ponto-cerebellar pathways, through which telencephalic areas convey to the cerebellum information on the emotionally competent stimuli along with context-related information; blue arrows = the cerebellum modulates the profile, intensity, and duration of the emotional responses in accordance with the context of the triggering stimulus by providing input to the induction and effector sites; structural and functional abnormalities in these sites may trigger emotional responses (pathologic tearfulness) that are contextually inappropriate.

In line with our hypotheses, we found correlates of pathologic tearfulness in the anterior hippocampus, the posterior cingulate cortex, the ventral pons, and the neocerebellum. Whether these abnormalities result directly from the acute, primary pathology of aLE or occur subsequently as a form of functional diaschisis or as a consequence of Wallerian degeneration^23^ remains to be determined (see also discussion in reference 12). Figure 4 summarizes the insight our study provides on the impairments underlying pathologic tearfulness in aLE, based on the model in reference 7.

### Anterior hippocampal volume

Right anterior hippocampal atrophy was associated with pathologic tearfulness. The right anterior hippocampal volume correlated across patients with scores for labile crying (CNS-LS), and patients with pathologic tearfulness showed less volume in the right anterior hippocampus in a voxel-wise whole-brain analysis. That hippocampal lesions should be associated with pathologic tearfulness is consistent with the involvement of limbic circuitry in emotion processing,^17^ especially with the relationship between recurrent stress and hippocampal damage in nonhuman primates,^19^ as well as with hippocampal pathology in psychiatric disorders.^43^ In particular, the primate anterior hippocampus is the homologue of the rodent ventral hippocampus, which plays a role in negative affect, by virtue of its connectivity with the amygdala and the hypothalamus.^18^ However, manually delineated hippocampal volumes did not differ between patients with and those without pathologic tearfulness, suggesting that atrophy may be confined to specific regions within the anterior hippocampus, a possibility that could be explored using subfield volumetry in future studies.

### Hippocampal dysconnectivity with the posteromedial cortex

Scores for labile crying (CNS-LS) strongly correlated with patients' reduced right hippocampal rsFC with the ventral posteromedial cortex (posterior cingulate, retrosplenial cortex, and precuneus). Evidence from functional neuroimaging of healthy adults supports a role of this region in empathic concern for emotional suffering and admiring virtue.^44^ Aberrant perspective taking and empathy has also been reported in hippocampal patients.^45,46^

### Pontocerebellar abnormalities

Volume reduction was also noted for patients with pathologic tearfulness in posterior portions of the right hemispheric cerebellar lobules VI/VIIa Crus I. These regions are embedded within the default mode network, which is fundamental for self-referential cognition.^47^ The cerebellum receives input from the basilar pons, and disruption of cortico-ponto-cerebellar circuits may lower the threshold for emotional expression.^7^ Moreover, anatomical and electrophysiologic work has recently disclosed evidence for cerebellar (lobules VI/VIIa Crus I)–hippocampal interactions, possibly via the pons.^25^ Consistent with these accounts, patients with pathologic tearfulness demonstrated aberrantly increased rsALFF in the ventral pons. The ventral pons relays input to the cerebellum from cortical regions including the parietal association cortices,^48^ where these patients showed reduced right hippocampal rsFC. Interestingly, pontine hemodynamic hyperactivity has been reported previously in a single case study of pathologic laughing,^6^ consistent with earlier reports of pathologic crying in cases of pontine myelinosis.^8^

### Abnormalities in the inferior parietal lobule, fusiform, and middle frontal gyri

Beyond the relationships that we had hypothesized, we also observed a series of unpredicted abnormalities associated with pathologic tearfulness: GM volume reduction and aberrantly increased rsALFF in the left fusiform gyrus, reduced rsALFF in the right inferior parietal lobule, and reduced rsFC between the right hippocampus and the right middle frontal gyrus. While activations in all of these regions have been repeatedly shown in self-face processing,^49,50^ the aberrantly increased rsFC and rsALFF in patients with pathologic tearfulness require further investigation, as they may reflect compensatory or maladaptive mechanisms.

Our study describes a novel disorder of emotion regulation following aLE that is characterized by residual pathologic tearfulness, is not related to low mood or cognitive impairment, and is associated with specific abnormalities within networks supporting emotion regulation. Clinicians need to be aware of the potential for such symptoms to develop after aLE and of the distress they can cause. Furthermore, pathologic tearfulness offers a useful neuropsychological model for exploring the neural mechanisms of emotion regulation and may provide insight into the breakdown of these mechanisms across a wide range of neurologic conditions. This will inform the development and refinement of behavioral and pharmaceutical interventions.

## Glossary

aLE: autoimmune limbic encephalitis
ANOVA: analysis of variance
BA: Brodmann area
BIS: Barratt Impulsiveness Scale
BOLD: blood oxygenation level–dependent
CBS: Cambridge Behaviour Scale
CNS-LS: Center for Neurologic Study–Lability Scale
DARTEL: diffeomorphic anatomical registration through the exponentiated lie algebra
EPI: echoplanar imaging
FWE: family-wise error
FWHM: full width at half maximum
GM: gray matter
HADS: Hospital Anxiety and Depression Scale
HC: healthy control
IQR: interquartile range
IRQ: Irritability Questionnaire
MAP: Memory and Amnesia Project
MNI: Montreal Neurologic Institute
MTL: medial temporal lobe
MVPA: multivariate pattern analysis
OPTIMA: Oxford Project to Investigate Memory and Ageing
PCA: principal component analysis
rsALFF: resting-state abnormalities in the local amplitude of low-frequency fluctuations
rsFC: resting-state functional connectivity
rsfMRI: resting-state fMRI
TIV: total intracranial volume
VBM: voxel-based morphometry
WM: white matter
